# Proteomic fingerprinting in HIV/HCV co-infection reveals serum biomarkers for the diagnosis of fibrosis staging

**DOI:** 10.1371/journal.pone.0195148

**Published:** 2018-04-02

**Authors:** Makan Golizeh, Carlos E. Melendez-Pena, Brian J. Ward, Sahar Saeed, Cynthia Santamaria, Brian Conway, Curtis Cooper, Marina B. Klein, Momar Ndao, on behalf of the Canadian Co-Infection Cohort (CTN222)

**Affiliations:** 1 Program in Infectious Diseases and Immunity in Global Health, The Research Institute of the McGill University Health Centre, Montreal, Quebec, Canada; 2 Division of Experimental Medicine, McGill University, Montreal, Quebec, Canada; 3 Division of Infectious Diseases and Chronic Viral Illness Service, McGill University Health Centre, Montreal, Quebec, Canada; 4 Vancouver Infectious Diseases Center, Vancouver, British Columbia, Canada; 5 The Ottawa Hospital-General Campus, Ottawa, Ontario, Canada; 6 Department of Microbiology and Immunology, McGill University, Montreal, Quebec, Canada; 7 National Reference Centre for Parasitology, The Research Institute of the McGill University Health Centre, Montreal, Quebec, Canada; Harvard Medical School, UNITED STATES

## Abstract

**Background:**

Hepatic complications of hepatitis C virus (HCV), including fibrosis and cirrhosis are accelerated in human immunodeficiency virus (HIV)-infected individuals. Although, liver biopsy remains the gold standard for staging HCV-associated liver disease, this test can result in serious complications and is subject to sampling errors. These challenges have prompted a search for non-invasive methods for liver fibrosis staging. To this end, we compared serum proteome profiles at different stages of fibrosis in HIV/HCV co- and HCV mono-infected patients using surface-enhanced laser desorption ionization time-of-flight mass spectrometry (SELDI-TOF MS).

**Methods:**

Sera from 83 HIV/HCV co- and 68 HCV mono-infected subjects in 4 stages of fibrosis were tested. Sera were fractionated, randomly applied to protein chip arrays (IMAC, CM10 and H50) and spectra were generated at low and high laser intensities.

**Results:**

Sixteen biomarkers achieved a p value < 0.01 (ROC values > 0.75 or < 0.25) predictive of fibrosis status in co-infected individuals and 14 in mono infected subjects. Five of these candidate biomarkers contributed to both mono- and co-infected subjects. Candidate diagnostic algorithms were created to distinguish between non-fibrotic and fibrotic individuals using a panel of 4 biomarker peaks.

**Conclusion:**

These data suggest that SELDI MS profiling can identify diagnostic serum biomarkers for fibrosis that are both common and distinct in HIV/HCV co-infected and HCV mono-infected individuals.

## Introduction

Morbidity and mortality in human immunodeficiency virus 1 (HIV)-infected individuals have significantly decreased due to the effective long-term combination antiretroviral therapy (cART) [1]. New complications have however emerged as key issues within this population. HIV/ Hepatitis C virus (HCV) co-infection, for instance, affects more than 30% of HIV-infected patients in developed countries. Although the impact of HCV on HIV disease progression is minimal, it is known that HIV accelerates HCV-related liver disease [2, 3]. The effects of HIV on HCV infection include higher rate of viral persistence and increased HCV viral loads (VL). Studies have shown that HCV–associated liver diseases such as fibrosis, cirrhosis and end stage liver disease (ESLD) are accelerated in HIV-infected individuals [4–7]. The mechanisms underlying rapid liver disease progression in HIV/HCV co-infected patients are likely multifactorial and are presently not completely understood.

Hepatic fibrosis results from the deposition of scar tissue and may lead to cirrhosis. It is characterized by distortion of the liver architecture and the major determinant of morbidity and mortality in the patients with liver disease [8]. In many countries the degree of hepatic fibrosis is the principal determinant to access highly efficacious HCV treatment, known as direct acting antivirals [9]. Liver biopsy remains the gold standard for staging HCV-associated liver disease [10, 11]. However, liver biopsy can result in serious complications, is costly and not feasible to repeat serially, and is subject to sampling error [12]. These problems have prompted a search for non-invasive methods for liver fibrosis staging such as Fibroscan, a transient elastography technology which is based on the assessment of liver stiffness [13]. Several factors can limit this examination such as morbid obesity, ascites, and small intercostal spaces and better result are shown in patients with severe fibrosis [8, 14]. Safe, reliable and simple alternatives are needed to diagnose and monitor fibrosis caused by HCV infection.

Proteomic fingerprinting is a diagnostic concept based on the idea that disease states are often associated with distinctive configurations of circulating proteins. Analysis of combinations of several biomarkers offers the possibility of enhanced diagnostic accuracy compared to individual biomarkers that have limited diagnostic sensitivities and specificities. Surface-enhanced laser desorption ionization-time-of-flight mass spectrometry (SELDI-TOF MS) offers high-throughput protein profiling of native biological specimens. This platform has been successfully used as a discovery tool for biomarkers associated with inflammation [15], cancers [16–18] and human infectious diseases [19–22]. SELDI-TOF MS serum profiling has accurately distinguished patients with different stages of liver disease, specifically those associated with HCV infections ranging from chronic hepatitis to HCV-associated hepatocellular carcinoma (HCC) [23].

To this end, we compared serum proteome profiles at different stages of fibrosis in HIV/HCV co- and HCV mono-infected patients using SELDI-TOF MS. Our aim was to identify a proteomic fingerprint that could be used to develop a diagnostic test to detect and stage liver fibrosis in HIV/HCV co-infected individuals. We report a SELDI-TOF MS-based alternative assay that can achieve high sensitivity and specificity for the detection of fibrosis in the patients with HIV/HCN co-infection.

## Materials and methods

### Study design, setting and population

The Canadian Co-infection Cohort Study (CCC, CIHR Canadian HIV Trials Network (CTN222)) is a prospective multicentre study recruiting HIV/HCV co-infected patients from 18 centres across Canada since 2003 with approval by participating research ethics boards described in details elsewhere [24]. As of October 2011, 1090 patients were enrolled. For this analysis participants were selected based on the availability of a serum specimen within one year of liver biopsy. HCV mono-infected patients undergoing liver biopsies were prospectively recruited from 3 sites participating in the CCC and serum samples were obtained within a year of liver biopsy. A total of 151 individuals were studied, including 68 HCV mono-infected and 83 HIV/HCV co-infected subjects. Patients at each of the 4 stages of fibrosis stage: F0-1 (F1), F2, F3, F4/ESLD (F4), as determined by liver biopsy, were selected for this analysis.

### Serum fractionation

Serum proteins were fractionated prior to the SELDI-TOF MS analysis as described [20]. Briefly, samples were fractionated on re-hydrated Q HyperD F beads by pH into 6 fractions using Bio-Rad (Hercules, CA) serum fractionation kit following manufacturer’s instructions. All of the steps of protein fractionation including binding and washing were performed on a BioMek 2000 laboratory automation workstation (Beckman Coulter, Fullerton, CA) with an integrated microplate shaker (MicroMix; Diagnostic Products Company, Los Angeles, CA) that holds an array bioprocessor (Bio-Rad). Serum fractions were stored at -20°C until analyzed. For quality control purposes, commercial human reference sera from healthy donors (Valley Biomedical, Winchester, VA) and duplicates were included in all the test runs.

### ProteinChip binding and mass spectrometry analysis

Samples were randomized within and across arrays with blank spots included as negative controls and applied to three types of ProteinChip arrays: weak cation exchange (CM10), immobilized metal affinity capture (IMAC30) and hydrophobic/reverse-phase (H50) (all from Bio-Rad). Arrays were prepared as previously described [25] and analyzed in a ProteinChip biology system reader (series PCS 4000) using the ProteinChip software version 3.5 (Bio-Rad). In a preliminary study (data not shown), a few samples from each group were bound onto a CM10 ProteinChip array and all the 6 fractions were analyzed. The fractions yielding the most satisfactory results (i.e. the most differentiating biomarkers) were selected for SELDI-TOF MS analysis: fractions 1 (pH 9 and flow through), 3 (pH 5) and 6 (organic) for analysis on CM10 arrays. Optimization experiments with IMAC30 and H50 arrays led to similar results. Therefore, to minimize the cost and time of analysis fractions 2, 4 and 5 were not subjected to the MS analysis.

Each spot was read at low- and high-energy laser intensities. Using external calibration standards (bovine insulin, 5,733.6 Da; ubiquitin, 8,564.84 Da; cytochrome *c*, 12,230.9 Da; β-lactoglobulin, 18,363.3 Da; horseradish peroxidase, 43,240 Da; and IgG, 147,300 Da), all spectra were subjected to mass calibration based on the settings used to collect the data. The baseline was subtracted using a setting of 15 times the expected peak width. Noise was subtracted at 2,000 Da for low-energy and at 10,000 Da for high-energy acquisition. All data were normalized by total ion current for either low intensity (2 to 100 kDa) or high intensity (10 to 200 kDa) using an external coefficient of 0.2. Spectra with normalization factors of more than double the mean were deleted. Analyses were performed in two steps. First, automated peak detection was applied, using cluster features of Biomarker Wizard software (Bio-Rad). Cluster with p values <0.05 (Mann-Whitney U test) were visually inspected, followed by manual peak relabeling. After relabeling, exact p values for differences in average peak intensity between two groups were calculated (Wilcoxon exact test): 1) negative population with F1 (no/mild fibrosis), 2) positive population with F3-F4 (significant fibrosis). Peaks with p values < 0.05 and receiver operating characteristic (ROC) values > 0.75 or < 0.25 were considered as potential biomarkers. Biomarker Pattern software (Bio-Rad) analysis was applied to the cleaned cluster data. This program uses a supervised pattern classification method (classification and regression tree (CART)) to identify peaks with the greatest contribution to discrimination between groups. The CART procedure seeks to minimize a cost function that balances prediction errors in either sense and the total number of biomarkers used. The relative importance of each peak in any given algorithm is measured by the order in which it is selected in the decision tree and the number of correct predictions credited to it. Similar analysis was performed to find discriminator peaks between the four stages of fibrosis (F0-1, F2, F3 and F4).

### Protein identification

Two samples that showed highest intensity in SELDI-TOF MS analysis for each biomarker were selected as positive samples and two with the lowest intensity were selected as negative controls. Enriched fractions were purified in a NuPAGE precast gel (Invitrogen Life Technologies, Carlsbad, CA) (180 V, 50 min, Tris buffer pH 8.2), stained with Colloidal Blue (Invitrogen) and the bands of interest were excised for in-gel digestion based on a protocol previously described [26]. Briefly, gel-bound proteins were denatured using dithiothreitol (10 mM in 100 mM ammonium bicarbonate, 25°C, 30 min), alkylated with 2-iodoacetamide (55 mM in 100 mM ammonium bicarbonate, 25°C, 30 min) and digested by trypsin (13 ng/ml in 10 mM ammonium bicarbonate with 10% acetonitrile, 37°C) overnight. Tryptic digests were extracted with 50:25:15:10 formic acid/acetonitrile/isopropanol/water (2 times) and 100% acetonitrile (2 times) and vacuum dried. Dried peptide samples were re-suspended in 0.1% trifluoroacetic acid (TFA), sonicated for 10 min and desalted with C18 reverse-phase ZipTip according to the manufacturer’s protocol (Millipore, Billerica, MA). Samples were eluted in 4 μL of α-cyano-4-hydroxycinnamic acid (CHCA) matrix (10 mg/mL in 50:50 acetonitrile/0.1% TFA in water). The solution was directly spotted onto a 384-well AB OptiTOF stainless steel plate (AB Sciex, Framingham, MA) and allowed to dry at room temperature.

Matrix-assisted laser desorption ionization time-of-flight mass spectrometry (MALDI-TOF MS) data were acquired on a 4800 Plus MALDI TOF/TOF Analyzer (AB Sciex) with the 4000 Series Explorer v3.5.3 software. Internal calibration was carried out using des-Arg-1-bradykinin (monoisotopic mass 904.4681), angiotensin I (1296.6853), glu^1^-fibrinopeptide B (1570.6774), adrenocorticotropic hormone (ACTH) fragments [1–17] (2093.0867), [18–39] (2465.1989) and [7–38] (3657.9294). In the positive-ion reflector mode, MS data were collected over a mass range of 800–4000 Da using a fixed laser intensity of 3200 nJ for 1000 shots/spectrum, with a uniformly random spot search pattern. In each MS spectrum, the 20 most abundant MS peaks were selected for MS/MS using an acquisition method that excluded ions with S/N less than 20. The precursor ions with the strongest S/N were acquired first using a 1 kV MS/MS operating mode in which the relative precursor mass window was set at 50 and the metastable suppression enabled. MS/MS acquisition of selected precursors was set to 2000 shots per spectrum with 50 shots per sub-spectrum using a fixed laser intensity of 4200 nJ.

Protein identification was performed with ProteinPilot 4.0.8 software using the Paragon algorithm (AB Sciex). Peptides present in positive samples but absent in the negative samples were selected. MS/MS data were searched against the UniProtKB/Swiss-Prot database (downloaded on March 7, 2017) for *Homo sapiens*, HCV and HIV. Trypsin was selected as the digestion enzyme. Other search parameters included cysteine alkylation by iodoacetamide, gel-based thorough ID with a focus on biological modifications. The mass spectrometry proteomics data have been deposited to the ProteomeXchange Consortium via the PRIDE [27] partner repository with the dataset identifier PXD009007.

## Results

Demographic and clinical characteristics of the study population are shown in Table 1. Co-infected and mono-infected patients were similar in most respects, except there was a higher proportion reporting recent alcohol use (63% vs. 38%) and fewer women (16% vs. 31%) among co-infected patients. Co-infected individuals were generally younger (mean 45.4 years) compared to mono-infected (mean 49.6 years) and had been HCV-infected for a shorter period of time (16.5 vs. 20.7 years). These last differences would have been expected knowing the accelerated progression of fibrosis in co-infected patients [2, 3]. Each group was divided into four categories according to liver biopsy score: F1 (n = 50), F2 (n = 49), F3 (n = 50) and F4 (n = 22) based on the Batts-Ludwig scoring system. The characteristics according to fibrosis score are also shown in Table 1.

**Table 1:** Demographic information on the patients with HCV mono-infection and HIV/HCV co-infection.

	HCV mono (n = 68)	Co-infection (n = 83)	P value	HCV mono-infection				HIV/HCV co-infection			
				F1 n = 20	F2 n = 20	F3 n = 20	F4 n = 8	F1 n = 30	F2 n = 19	F3 n = 20	F4 n = 14
**Age (yr)**	49.59± 0.94	45.39± 0.87	<0.01	46.95± 1.90	50.25± 2.16	50.95 ± 1.20	51.13 ± 1.63	44.63± 1.69	44.72± 1.76	45.63 ± 1.66	47.93 ±1.81
**Sex**											
men	69%	82%	<0.05	60%	70%	75%	63%	88%	78%	84%	64%
women	31%	16%		35%	30%	25%	37%	8%	22%	11%	36%
transgender	0%	2%		5%	0%	5%	0%	4%	0%	5%	0%
**Ethnicity**											
Caucasian	82%	82%	1	75%	80%	75%	100%	81%	83%	79%	86%
other	18%	18%		25%	20%	25%	0%	19%	17%	21%	14%
**Alcohol**	88%	95%	0.178	55%	65%	65%	75%	100%	83%	100%	100%
Last 6 month	38%	63%	<0.01	40%	35%	40%	25%	74%	56%	63%	58%
**Duration of HCV (yr)**	20.75 ± 1.81	16.53± 1.12	<0.05	19.49 ± 3.18	17.49± 3.75	23.64 ± 3.36	25.37± 4.09	14.46 ± 1.83	13.64± 2.11	20.46 ± 2.49	19.58± 2.58
**Duration of HIV (yr)**	**-**		**-**	**-**	**-**	**-**	**-**	11.37 ± 1.15	10.47 ± 1.59	14.69 ± 1.74	12.59 ± 1.57
**CD4 count**	**-**		**-**	**-**	**-**	**-**	**-**	520.6±47.12	410.8±39.87	501.4±39.37	484.9±65.14
**VL**	**-**		**-**	**-**	**-**	**-**	**-**	992.7±661	*1880±1498	47.21±1.047	44.07±2.414

HCV: hepatitis C virus; HIV: human immunodeficiency virus; VL: viral load. Values are median (SE) or %;

* p < 0.05;

### Comparison of SELDI-TOF MS spectra in HIV/HCV-co infection and HCV mono-infection cohorts

**Fig 1:**
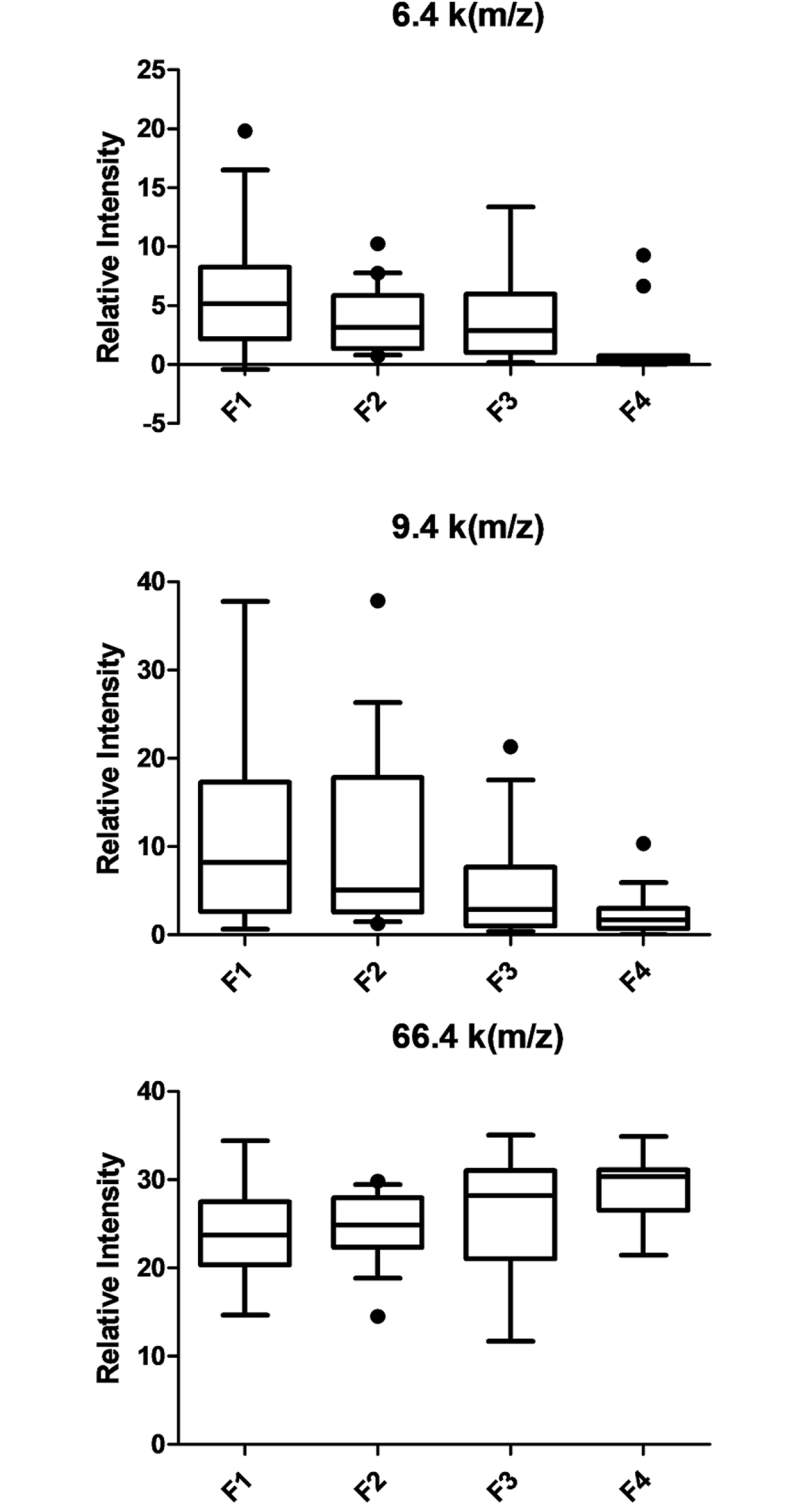
Serum levels of the 6.4, 9.3 and 66.6 k(m/z) biomarkers in HIV/HCV co-infected patients according to fibrosis stage. Box–whiskers: 10–90 percentile.

The SELDI-TOF MS approach was applied to samples from 151 patients. Due to the difference in parameters (age and duration of HCV infection) between HCV mono-infected and HIV/HCV co-infected individuals, the spectra of each group were analyzed separately. Most biomarker programs focus on a single disease and have a dichotomous pattern (i.e. either positive or negative). Our primary comparison was non-fibrotic patients (F1) to more advanced fibrotic patients (F3-F4). Due to the relatively small number of samples in F4 group we pooled F3 and F4 groups together for analysis. Using Biomarker Wizard software, sixteen potential biomarkers with significant intensity difference and good area under curve (AUC) from ROC curve analysis were identified (Table 2). Six of these individual biomarkers were down-regulated in the group with advanced fibrosis, while eight were up-regulated with moderate changes between the two groups (1.33 to 2.55-fold). When we tested the ability of these 14 biomarkers to differentiate patients with regards to their stage of liver fibrosis (F1, F2, F3 and F4), 8 out of 14 had significant intensity differences with a p value < 0.01. The three biomarkers demonstrating the most significant difference in expression (6.4, 9.3 and 66.6 k(m/z)) are shown in Fig 1. The cumulative data illustrate how changes in multiple protein biomarkers can be used as “fingerprints” reflective of a particular disease state. Similar analysis was performed with HCV mono-infected samples. Fourteen classifiers with significant intensity differences between F1 and F3-4 were identified (S1 Table). Representative SELDI-MS spectra of the identified biomarkers can be found under S1 Fig. SELDI-TOF MS peak values are available in S1 File.

**Table 2:** SELDI-TOF MS biomarkers detected in HIV/HCV co-infection.

m/z (/1,000)	Fractions and chemistries	P value (F1 vs F3-4)	AUC for ROC Curve (fold F1/F3-4)	Mean signal intensity ± SE				P value
				F1 (n = 20)	F2 (n = 20)	F3 (n = 20)	F4 (n = 8)	
**2.2**	F6 CM10	0.002	0.73(1.65)	6.45 ± 2.54	7.19 ± 2.68	9.05 ± 3.01	12.20±2.61	0.008
**4.6**	F3 IMAC30	0.006	0.31(-2.04)	4.05 ± 2.01	2.68 ± 1.64	2.44 ± 1.56	1.09±1.36	0.010
**6.4**	F3 H50	0.007	0.27(-2.14)	6.42 ± 3.05	3.75 ± 2.94	3.97 ± 2.45	1.61±1.62	0.002
**8.1**	F6 CM10	0.007	0.27(-0.58)	3.07 ± 1.83	2.97 ± 1.45	2.04 ± 1.06	1.51±1.01	0.006
**8.9**	F3 IMAC	0.005	0.31(-1.71)	1.82 ± 1.19	1.59 ± 1.13	1.23 ± 1.38	0.73±0.82	0.013
**9.3**	F6 H50, F3 IMAC30-CM10	0.002	0.29(-2.55)	11.33 ± 3.37	10.12 ± 3.18	5.84 ± 2.42	2.50±1.68	0.002
**13.8**	F6-F1 H50,	0.004	0.30(-1.33)	1.80 ± 0.54	1.39 ± 0.67	1.45 ± 0.33	1.14±0.46	0.004
**18.4**	F1 CM10	0.009	0.28(2.13)	0.29 ± 1.19	0.46 ± 1.00	0.11 ± 1.00	0.17±0.83	0.021
**22.8**	F6-F3 CM10	0.010	0.67(1.65)	0.60 ± 0.78	0.82 ± 0.91	0.74 ± 0.86	1.28±1.11	0.014
**24.2**	F1 CM10	0.018	0.71(1.74)	0.58 ± 0.76	0.85 ± 0.92	0.87 ± 0.93	1.13±0.85	0.071
**33.3**	F3 CM10-IMAC30	0.004	0.71(1.32)	3.21 ± 1.79	3.91 ± 1.98	4.21 ± 2.05	4.54±1.20	0.015
**66.6**	F3 IMAC30	0.003	0.71(1.16)	23.88 ± 4.89	24.60 ± 4.96	26.75 ± 5.17	29.25±1.92	0.002
**78.8**	F6 IMAC30	0.010	0.69(1.54)	0.45 ± 0.67	0.66 ± 0.81	0.61 ± 0.78	0.76±0.64	0.030
**133.4**	F3 IMAC30	0.006	0.71(1.14)	2.32 ± 1.52	2.38 ± 1.54	2.69 ± 1.64	2.67±0.74	0.015

Mass-over-charge (m/z) value, mean signal intensity, and area-under-curve (AUC) for selected individuals differentially expressed peptides/proteins between hepatic fibrosis 0–1 (F1), fibrosis 2 (F2), fibrosis 3 (F3) and ESLD or fibrosis 4 (F4) patients.

**Fig 2:**
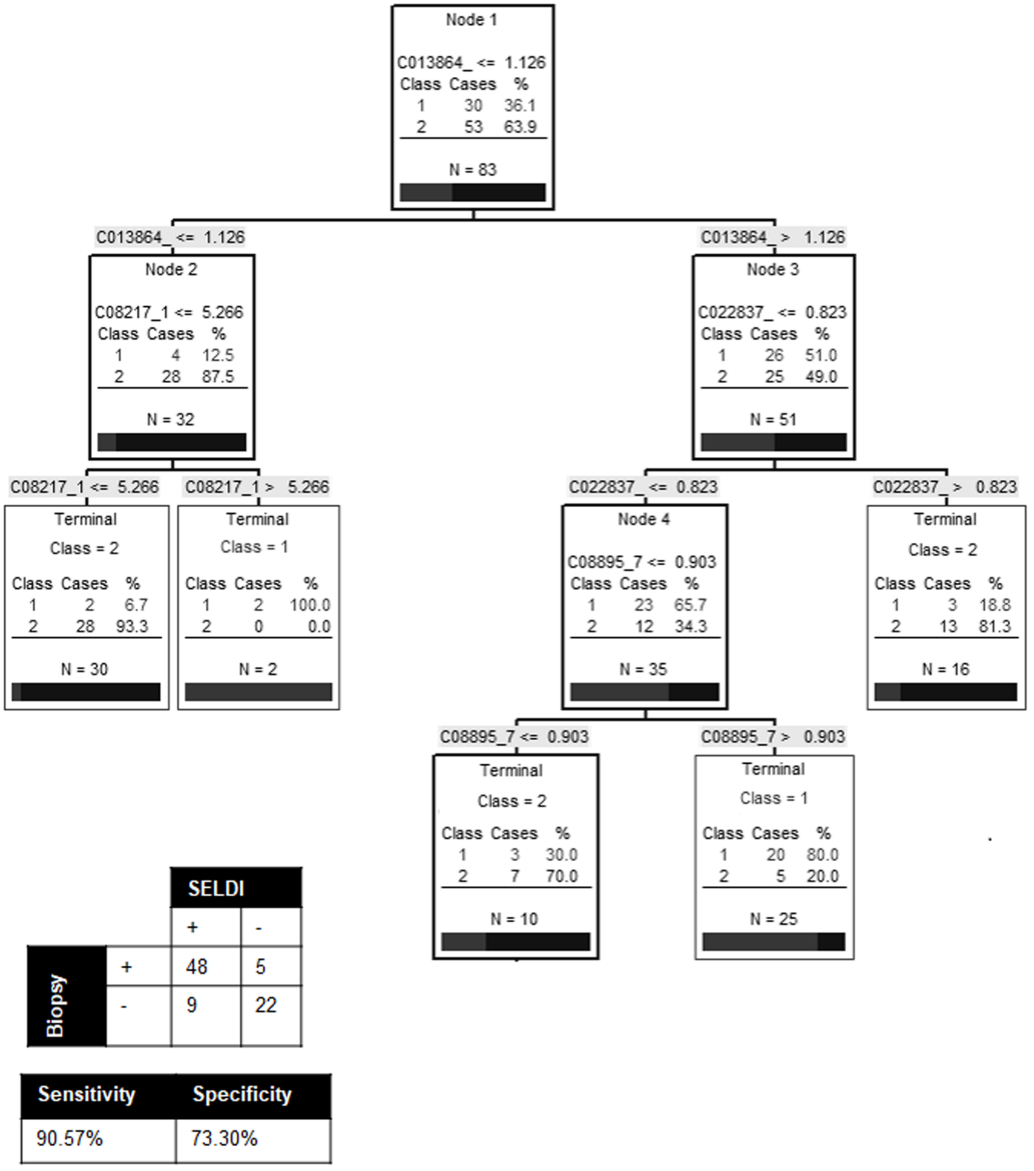
Decision tree to differentiate non-fibrotic individuals (F1) from individuals with significant fibrosis (F2 and above) in HIV/HCV co-infection. Biomarker pattern based on CART analysis was used to generate candidate diagnostic algorithms. The numbers on the root node, the descendant nodes and the terminal nodes represent the mass values in followed by the intensity value. Non-fibrotic individuals = Class 1, individuals with significant fibrosis = Class 2.

To select biomarkers with the greatest discriminatory power to distinguish between non-fibrotic (F1) and fibrotic patients (F2-3-4), the biomarker pattern recognition software was used to generate random training and test data sets and candidate decision trees. In co-infected patients, 4 biomarkers (8.1, 8.9, 13.8 and 22.8 k(m/z)) served as the main splitters with the highest individual predictive rates and were used to build an algorithm. A representative decision tree that achieved 90.6% sensitivity and 73.3% specificity is presented in Fig 2. This algorithm was able to correctly diagnose 77/83 individuals (92.8%). However, when the same algorithm was applied to the HCV mono-infected samples, we obtained an accuracy of only 53% (35/64), 50% sensitivity and 55% specificity (data not shown). To evaluate the possible influence of HIV disease activity on these biomarkers, we assessed the correlation between these biomarkers and the markers of HIV progression (HIV RNA and CD4 counts). Except for the peak at 22.8 k(m/z), which was correlated with HIV RNA, no other correlation was observed (S2 Fig). Using the same 4 biomarkers we created a new algorithm to try to differentiate individual states of liver fibrosis (F1, F2, F3 and F4) in co-infected individuals. The decision tree only achieved 59.0% (49/83) accuracy demonstrating that our algorithms can quite reliably diagnose patients with significant fibrosis (*vs*. non-fibrotic) but are much less accurate for distinguishing between the middle stages of fibrosis.

**Fig 3:**
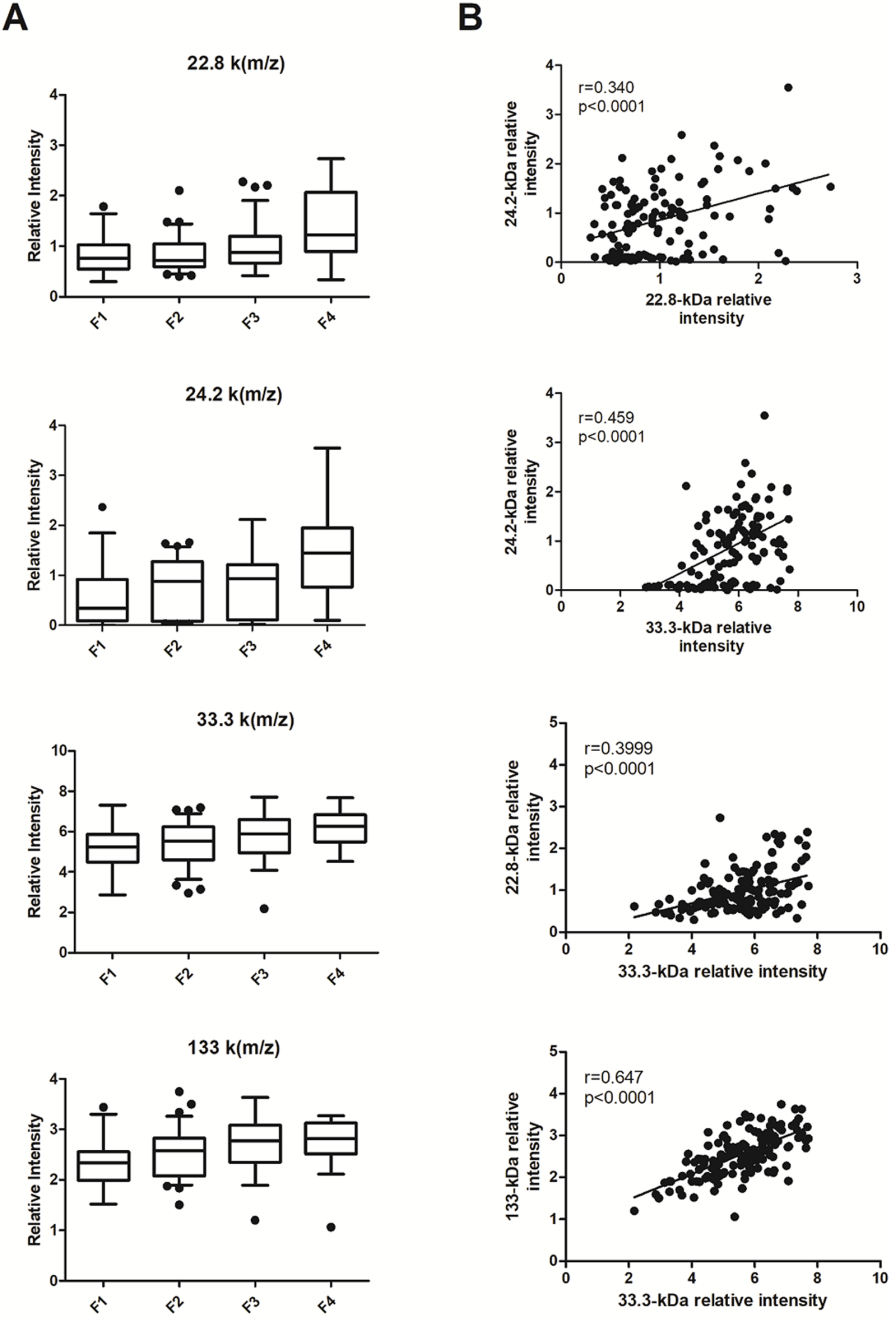
Serum levels of the 22.8, 24.2, 33.3 and 133.4 k[m/z] biomarkers for HIV/HCV co-infected and HCV mono-infected individuals combined according to fibrosis stage. (A) Box—whiskers; 10–90 percentile. (B) Spearman’s correlation between different biomarkers. Each point represents a separate individual.

Five biomarkers, 22.8, 24.2, 33.3, 66.6 and 133.4 k[m/z], were present in both mono- and co-infected individuals (Fig 3A) and were highly correlated with one another (Fig 3B). However, unlike the biomarkers specific to co-infection, we were not able to obtain good decision trees with these biomarkers (data not shown).

### Biomarker identification

Presumptive identifications were based on peptides present in the positive samples and absent in negative samples. Serum fractionations were run on sodium dodecyl sulfate-denaturing polyacrylamide gel electrophoresis (SDS-PAGE). Selected bands were cut with their negative control. Table 3 summarizes the biomarkers identified by MALDI-TOF MS when comparing positive and negative samples.

**Table 3:** Selected biomarkers of HCV mono- and HIV/HCV co-infection identified by SDS-PAGE/MALDI-TOF MS.

m/z (/1,000)	Protein name	Theoretical MW (kDa)
13.8	Ig kappa chain C region	11.6
18.4	Haptoglobin alpha chain	15.9
22.8	Apolipoprotein A1	30.7
46.8*	Haptoglobin	45.2
78.8	Serotransferrin	77.0
84.6*	Plasminogen	90.5

MW: molecular weight. Serum fractions were run on SDS-PAGE. Selected bands were cut with their negative control. Biomarkers that were significantly over- or under-expressed were identified using matrix-assisted laser desorption/ionisation-time-of-flight (MALDI-TOF) MS. Presumptive identification was based on peptide present in the positive sample and absent in negative sample

* = biomarkers that were significantly over- or under-expressed in mono-infection.

**Fig 4:**
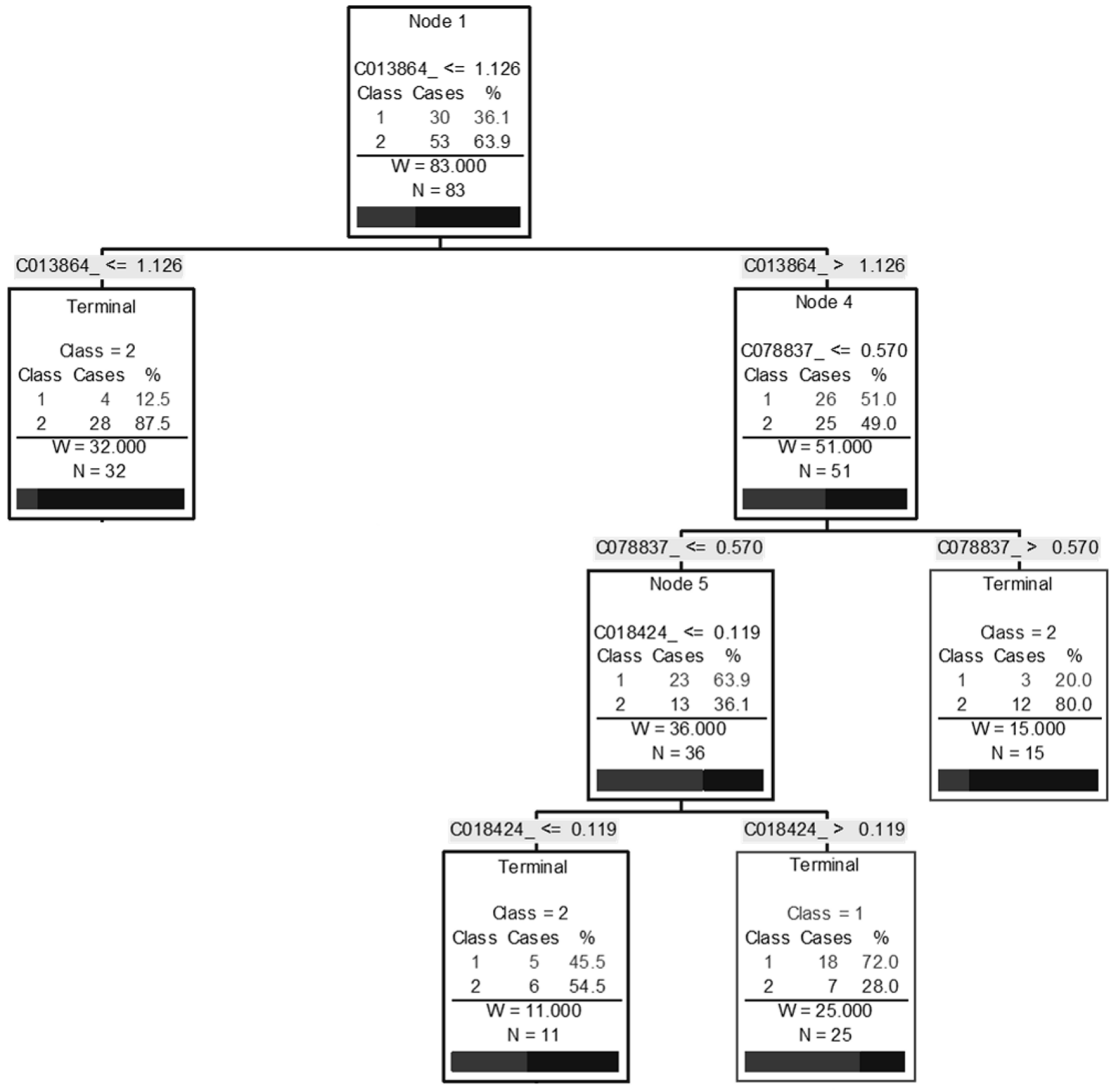
Decision tree to differentiate non-fibrotic individuals (F1) from individuals with significant fibrosis (F2 and above) in HIV/HCV co-infection using known biomarkers. A biomarker pattern based on CART analysis was used to generate candidate diagnostic algorithms. The numbers in the root node, the descendant nodes and the terminal nodes represent mass values (Cxxxxxx) followed by intensity values. Class 1: non-fibrotic individuals, Class 2: individuals with significant fibrosis.

**Fig 5:**
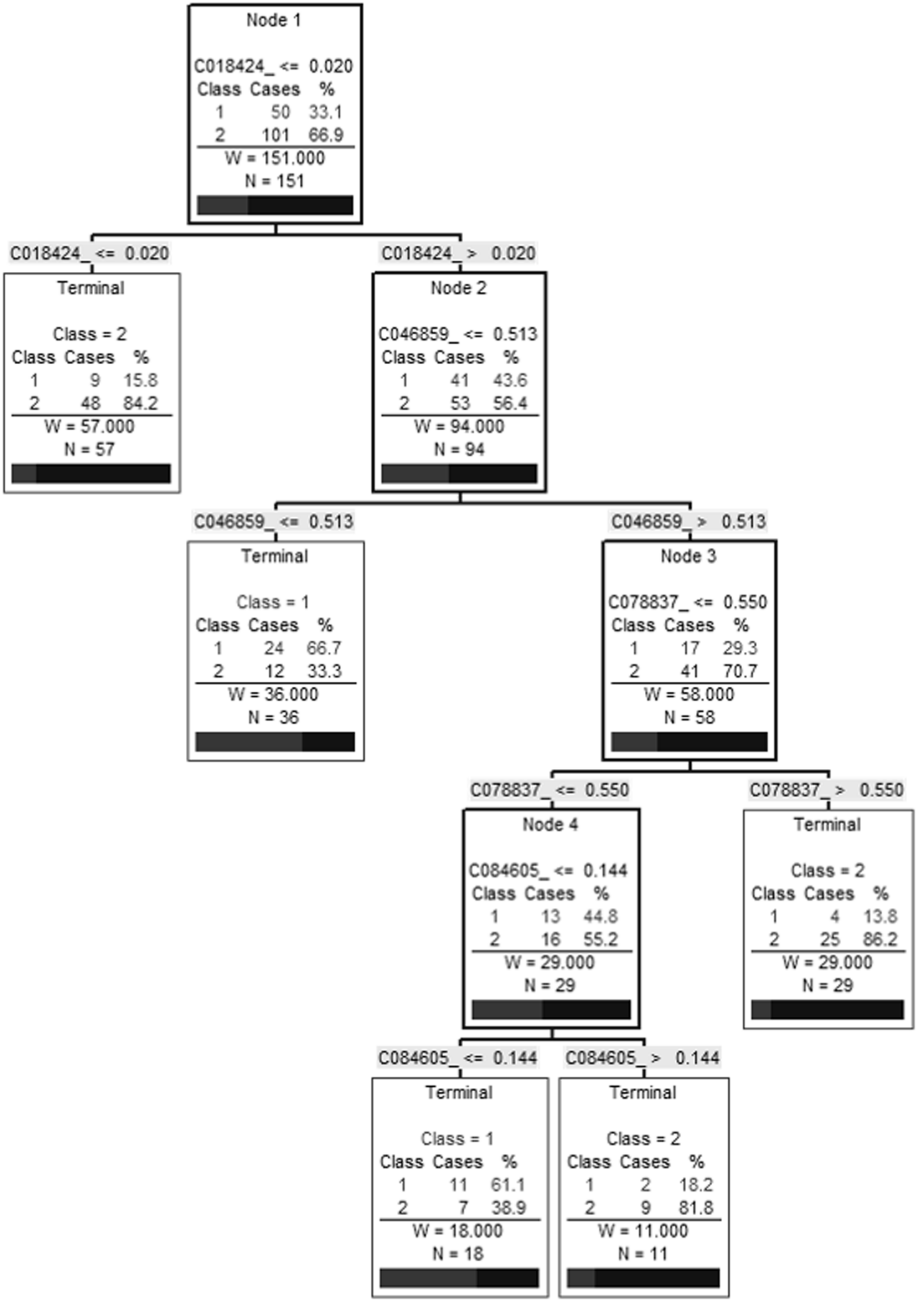
Decision tree to differentiate non-fibrotic individuals (F1) from individuals with significant fibrosis (F2 and above) in HCV-infected individuals using known biomarkers. A biomarker pattern based on CART analysis was used to generate candidate diagnostic algorithms. The numbers in the root node, the descendant nodes and the terminal nodes represent mass values (Cxxxxxx) followed by intensity values. Class 1: non-fibrotic individuals, Class 2: individuals with significant fibrosis.

All candidate biomarkers were of host origin and included: immunoglobulin (Ig) kappa chain constant (C) region (13.8 k(m/z)), haptoglobin (46.8 k(m/z)) and its alpha chain (18.4 k(m/z)), a possible truncated form of apolipoprotein A1 (ApoA1 (22.8 k(m/z)), plasminogen (84.6 k(m/z)) and serotransferrin (78.8 k(m/z)). Using the marker pattern recognition software, we tried to establish a decision tree using only these clusters to distinguish between fibrotic patients (F2, 3 and 4) and healthy individuals (F1) with co-infection. We obtained a 3-node algorithm using biomarkers at 13.8, 18.4 and 78.8 k(m/z). This algorithm was able to correctly diagnose 64/83 (77.1%) individuals and had a sensitivity and specificity of 86.8% and 60.0% respectively (Fig 4). A second analysis was performed with all 151 individuals and using these same known biomarkers and we obtained similar results with an efficiency of 77.4% (117/151) with a sensitivity of 81.1% and specificity of 70.0%. However, this algorithm had four nodes using the biomarkers at 18.4, 46.8, 78.8 and 84.6 k(m/z) (Fig 5).

## Discussion

To date, new biomarkers for fibrosis have been sought primarily by using differential two-dimensional gel electrophoresis (2D-GE) separations of tissues or serum together with mass spectrometry for protein identification [28, 29]. Proteomic analysis of plasma or serum derived from HCV-infected subjects is an emerging technique for the identification of biomarkers indicative of disease progression and severity [23, 30–33]. Using plasma samples from clinically defined patients of fibrosis progression by biopsy, we have obtained results that support the potential use of SELDI-TOF MS profiling as a tool to follow disease progression.

Using SELDI-TOF MS we observed changes in the level of 14 plasma proteins/peptides in mono-infected and 14 in co-infected during the progress of hepatic fibrosis. We obtained satisfactory decision tree with an accuracy of 92.8% to identify non-fibrotic patients compared individuals with significant fibrosis in co-infection (81.3% sensitivity and 90.6% specificity). Some “misclassified” patients in our algorithm could be due to the low reliability of the biopsy-assigned disease stage categorisation. We need to take into consideration that our reference point was the biopsy test done within the year of the sample collection and that biopsies are subject to sampling error and inter-observer variability. The aspirate aminotransferase (AST) to platelet ratio index (APRI) has been validated as a surrogate marker of significant hepatic fibrosis in HIV/HCV co-infection [34]. In support of this possibility, five out of eight co-infected patients “misclassified” by our SELDI-TOF MS biomarkers had APRI values that did not correlate with their biopsy score.

Unfortunately, we were unable to generate an algorithm that could accurately diagnose both mono- and co-infected using the same set of biomarkers. This could be due to the biological differences between the infections. Even though we observed the same phenotype (liver fibrosis), it is possible that active liver disease and its progression are different in the two populations. Several studies have reported that co-infected individuals progress more rapidly to ESLD than mono infected individuals [4–7]. Therefore, it is possible that biomarkers we observed in co-infection are, in fact, markers for accelerated fibrosis. The differences between the mono and co-infected protein fingerprint observed suggest that further analyses might shed light on underlying pathologic mechanisms for more accelerated liver fibrosis progression among HIV positive patients. This could be achieved by carefully matching samples tested by the important covariates that determine the risk for accelerated disease in HCV.

It is important to note that most of the biomarkers used in the algorithm in HIV/HCV co-infection, with the exception of one (22.8-k(m/z)), did not correlate with markers of HIV disease progression (e.g. HIV RNA or CD4 counts). Consequently, these biomarkers are probably related to liver fibrosis. They can reflect the immune response or inflammatory environment that is present during the disease. It is important to emphasize that, even though we have detected biomarkers that can be used to stage liver fibrosis in mono- and co-infected individuals, we cannot affirm that these biomarkers are specific for HCV-induced fibrosis. In order to establish specificity, we will need to validate our algorithms with non-HCV liver anomalies such as alcoholic liver disease, hepatitis B-induced fibrosis or HCC. It is very likely that several of our candidate biomarkers are ‘general’ markers for liver damage since most are liver-derived and some have already been found in liver infection [21]. In addition, we observed 5 biomarkers that were present in both mono and co-infection and had a good correlation between them. This leads us to believe that there is a potential to identify biomarkers that would be able to diagnose fibrosis independent of HIV infection status.

While several non-invasive models utilize tests that are not routinely available, some groups have incorporated routine tests and laboratory data to assess fibrosis non-invasively [35]. We tried to improve our algorithms by adding APRI values without success [data not shown]. Although Fibroscan has largely replaced liver biopsy clinically it still has poor overall specificity and lack of sensitivity in the middle stages of fibrosis. It might be of interest to evaluate the potential of incorporating Fibroscan to improve the accuracy of our diagnostic algorithms particularly for distinguishing the middle stages of fibrosis.

In the emerging field of MS-based protein profiling of body fluids, the profile itself can be used to diagnose disease. Extending its use does not depend on identification of the proteins in the discriminating peaks. However, protein identification is still possible, and adds biological relevance to the findings. We were able to identify, Ig kappa chain C region, haptoglobin, a truncated form of ApoA1 and plasminogen. It is interesting to observe that most of these proteins have already being identified as biomarkers for fibrosis. For example, the Fibrotest is based on haptoglobin, alpha-2 macroglobulin, gamma globulin, ApoA1, gamma glutamyl transferase and total bilirubin measurement in serum specimens [36]. Ig kappa C region is known to be a good biomarker for inflammation and malignancy [37], it is a predictor of non-Hodgkin’s lymphoma in patients with HIV [38] and patients with HCV [39]. Our data demonstrate that using SELDI-TOF MS technology we could stage discrete levels of liver fibrosis as changes of intensity of these biomarkers are detected. Even when we used peaks from already known markers, we weren’t able to obtain an optimal algorithm compare to the unidentified peaks.

The sera used in our study were fractionated without prior depletion of the most abundant serum proteins. High-abundance proteins can mask the presence of lower-abundance proteins of either host or virus origin in MS studies. Some of the identified biomarkers proved to be fragments of full length of abundant proteins. These fragments may be products of the natural course of protein degradation or associated with the extent and/or type of infection. Future work will focus on identifying less abundant biomarkers, which could be host related or virally derived. The identification of a viral derived plasma biomarker could greatly enhance the specificity of our assay.

Other biomarkers have been identified in HIV/HCV mono and co-infected individuals such as POTE ankyrin domain family, member E, histidine-rich glycoprotein, fibronectin and complement C3 [40]. As some have similar mass as the biomarkers we discovered by SELDI-TOF MS, it would be of interest to confirm these using immunocapture assay. In addition, Yang *et al* [41] found complement C4-a and inter-alpha-trypsin inhibition to heavy chain H4, as biomarker candidates for predicting hepatic fibrosis using Isobaric Tags for Relative and Absolute Quantitation (iTRAQ) and differential gel electrophoresis (DIGE). The authors also found ApoA1, Ig kappa chain C region, haptoglobin and others as potential biomarker candidates. However, this study only included pooled serum samples of 24 people with chronic HCV and 6 non-infected individuals. Using SELDI-TOF MS serum profiling, Schwelgle *et al* [23] found 4 biomarkers (5.8, 8.9, 9.5 and 11.7 k(m/z)) that helped differentiate between healthy controls and HCC patients. In this study, they did not fractionate samples but they concentrated on low-mass proteins that have a metal-binding affinity. Our study differs significantly in that we only tested HCV infected individuals, we looked at high- and low-mass proteins after fractionation of the samples and we used 3 different types of ProteinChips (metal binding, weak cation exchange, and hydrophobic/reverse-phase).

Unlike previous studies using SELDI-TOF MS to diagnose a disease or infection, we aimed to evaluate the progression of a disease and not a dichotomous “positive” and “negative” outcome. As expected we did not observe either absence or presence of specific biomarkers/peaks but rather a gradual difference in plasma levels of certain peptides/proteins at the different stages of liver fibrosis. It would be interesting to see if by scaling each biomarker and adding to obtain a score, we could accomplish a better correlation with the stage of liver fibrosis in these patients. The CCC study has enrolled co-infected individuals since 2003. We are in the process of studying longitudinal changes of these biomarkers in co-infected individuals during the progression of fibrosis. In addition, we wonder if these biomarkers may vary after treatment of HCV infection as it has been shown with other markers [42, 43].

Non-invasive measures of liver fibrosis are gaining acceptance for long-term evaluation of hepatic complication in HIV/HCV co–infection [44]. We have investigated the serum proteome profile of HIV/HCV co-infected and HCV mono-infected patients by SELDI-TOF MS. The identification of the biomarkers may also give unique insight into the complex and prolonged host-virus interactions that lead to liver fibrosis.

## Supporting information

S1 Table SELDI-TOF MS Spectra in HCV mono-infection. (DOCX)

S1 File SELDI-TOF MS peak values. (XLSX)

S1 Fig Representative SELDI-MS spectra of the detected biomarkers from selected HIV/HCV co-infected patients with no/mild (F1) and significant (F3-4) liver fibrosis. (DOCX)

S2 Fig Biomarkers in HIV/HCV co-infection do not correlate with VL and CD4 count. (DOCX)
