# Supplementary material for: Proteomic fingerprinting in HIV/HCV co-infection reveals serum biomarkers for the diagnosis of fibrosis staging
Source: PLoS One. 2018 Apr 2;13(4):e0195148. doi: 10.1371/journal.pone.0195148 (PMC5880398; doi:10.1371/journal.pone.0195148)
Supplement: S1 Table — (DOCX) [file pone.0195148.s001.docx]

**S1 Table. SELDI-TOF MS Spectra in HCV mono-infection**. Mass (m/z), mean signal intensities, and area-under-curve (AUC) for selected individuals differentially expressed peptides/proteins between hepatic fibrosis 0-1 (F1), fibrosis 2 (F2), fibrosis 3 (F3) and ESLD or fibrosis 4 (F4) patients.

| **m/z (/1,000)** | **Fractions and chemistries** | **P value**  **( F1 vs F3-4)** | | | **AUC for ROC Curve (fold F1/F3-4)** | | | | **Mean signal intensity ± SE** | | | | | | | | | | | | | | | | **P value** | |  |
| --- | --- | --- | --- | --- | --- | --- | --- | --- | --- | --- | --- | --- | --- | --- | --- | --- | --- | --- | --- | --- | --- | --- | --- | --- | --- | --- | --- |
|  |  |  |  |  |  |  |  |  | **F1 (n=20)** | | | | **F2 (n=20)** | | | | **F3 (n=20)** | | | | **F4 (n=8)** | | | |  |  |  |
| **2.5** | F1 IMAC30 | 0.003 |  | 0.76 | | ( | 2.80 | ) | | 2.37 | ± | 1.54 | | 2.76 | ± | 1.66 | | 7.17 | ± | 2.68 | | 5.30 | ± | 2.51 | | 0.008 | |
| **4.1** | F1 CM10, F3-F6 H50 | 0.001 |  | 0.91 | | ( | 2.34 | ) | | 3.21 | ± | 1.79 | | 5.90 | ± | 2.43 | | 7.10 | ± | 2.66 | | 7.50 | ± | 1.32 | | 0.005 | |
| **4.6** | F1,F3 CM10-IMAC30 | 0.001 |  | 0.22 | | ( | -2.40 | ) | | 10.10 | ± | 3.18 | | 6.04 | ± | 2.46 | | 4.45 | ± | 2.11 | | 3.58 | ± | 1.57 | | 0.014 | |
| **9.3** | F3 IMAC30, F3 CM10 | 0.002 |  | 0.23 | | ( | -2.33 | ) | | 11.72 | ± | 3.42 | | 7.92 | ± | 2.81 | | 5.14 | ± | 2.27 | | 4.78 | ± | 2.55 | | 0.017 | |
| **18.4** | F1 CM10 | 0.011 |  | 0.30 | | ( | -5.86 | ) | | 0.54 | ± | 0.74 | | 0.04 | ± | 0.21 | | 0.12 | ± | 0.34 | | 0.01 | ± | 0.09 | | 0.052 | |
| **22.8** | F3 CM10 | 0.016 |  | 0.70 | | ( | 1.41 | ) | | 0.76 | ± | 0.87 | | 0.83 | ± | 0.91 | | 0.97 | ± | 0.99 | | 1.30 | ± | 0.80 | | 0.043 | |
| **24.2** | F1 CM10 | 0.048 |  | 0.68 | | ( | 2.47 | ) | | 0.51 | ± | 0.71 | | 0.73 | ± | 0.86 | | 1.03 | ± | 1.02 | | 1.98 | ± | 0.99 | | 0.013 | |
| **27.6** | F1 CM10 | 0.013 |  | 0.30 | | ( | -2.31 | ) | | 0.12 | ± | 0.35 | | 0.04 | ± | 0.21 | | 0.05 | ± | 0.23 | | 0.05 | ± | 0.26 | | 0.025 | |
| **33.3** | F3 IMAC30 | 0.013 |  | 0.70 | | ( | 1.15 | ) | | 5.31 | ± | 2.30 | | 5.35 | ± | 2.31 | | 6.02 | ± | 2.45 | | 6.23 | ± | 1.04 | | 0.067 | |
| **46.8** | F1 CM10 | 0.004 |  | 0.74 | | ( | 1.75 | ) | | 0.41 | ± | 0.64 | | 0.49 | ± | 0.70 | | 0.64 | ± | 0.80 | | 0.94 | ± | 0.63 | | 0.022 | |
| **66.6** | F3 IMAC30 | 0.001 |  | 0.75 | | ( | 1.16 | ) | | 24.62 | ± | 4.96 | | 26.05 | ± | 5.10 | | 28.36 | ± | 5.33 | | 29.34 | ± | 1.96 | | 0.015 | |
| **84.6** | F6 IMAC30 | 0.003 |  | 0.76 | | ( | -1.63 | ) | | 0.10 | ± | 0.32 | | 0.18 | ± | 0.42 | | 0.18 | ± | 0.42 | | 0.14 | ± | 0.23 | | 0.003 | |
| **100.2** | F3 IMAC30 | 0.004 |  | 0.77 | | ( | 1.51 | ) | | 0.16 | ± | 0.41 | | 0.21 | ± | 0.46 | | 0.24 | ± | 0.49 | | 0.26 | ± | 0.29 | | 0.036 | |
| **133.4** | F3 IMAC30 | 0.000 |  | 0.79 | | ( | 1.21 | ) | | 2.34 | ± | 1.53 | | 2.68 | ± | 1.64 | | 2.79 | ± | 1.67 | | 2.91 | ± | 0.51 | | 0.003 | |
