# Supplementary material for: Proteomic fingerprinting in HIV/HCV co-infection reveals serum biomarkers for the diagnosis of fibrosis staging
Source: PLoS One. 2018 Apr 2;13(4):e0195148. doi: 10.1371/journal.pone.0195148 (PMC5880398; doi:10.1371/journal.pone.0195148)
Supplement: S1 Fig — (DOCX) [file pone.0195148.s003.docx]

**S1 Fig. Representative SELDI-MS spectra of the detected biomarkers from selected HIV/HCV co-infected patients with no/mild (F1) and significant (F3-4) liver fibrosis**

**F1**

**F3-4**

**F1**

**F3-4**

**F1**

**F3-4**

**F1**

**F3-4**

**F1**

**F3-4**

**F1**

**F3-4**

**F1**

**F3-4**

**F1**

**F3-4**

**F1**

**F3-4**

**F1**

**F3-4**

**F1**

**F3-4**

**F1**

**F3-4**

**F1**

**F3-4**

**F1**

**F3-4**
