# Supplementary material for: Proteomic fingerprinting in HIV/HCV co-infection reveals serum biomarkers for the diagnosis of fibrosis staging
Source: PLoS One. 2018 Apr 2;13(4):e0195148. doi: 10.1371/journal.pone.0195148 (PMC5880398; doi:10.1371/journal.pone.0195148)
Supplement: S2 Fig — (DOCX) [file pone.0195148.s004.docx]

**S2 Fig.** **Biomarkers in HIV/HCV co-infection do not correlate with VL and CD4 count.** Each data point represent results from separate individuals showing the relative intensity of 8.2, 8.8, 13.8 and 22.8 k(m/z) and patient VL in A and CD4 counts in B. A spearman’s correlation test was used.

**
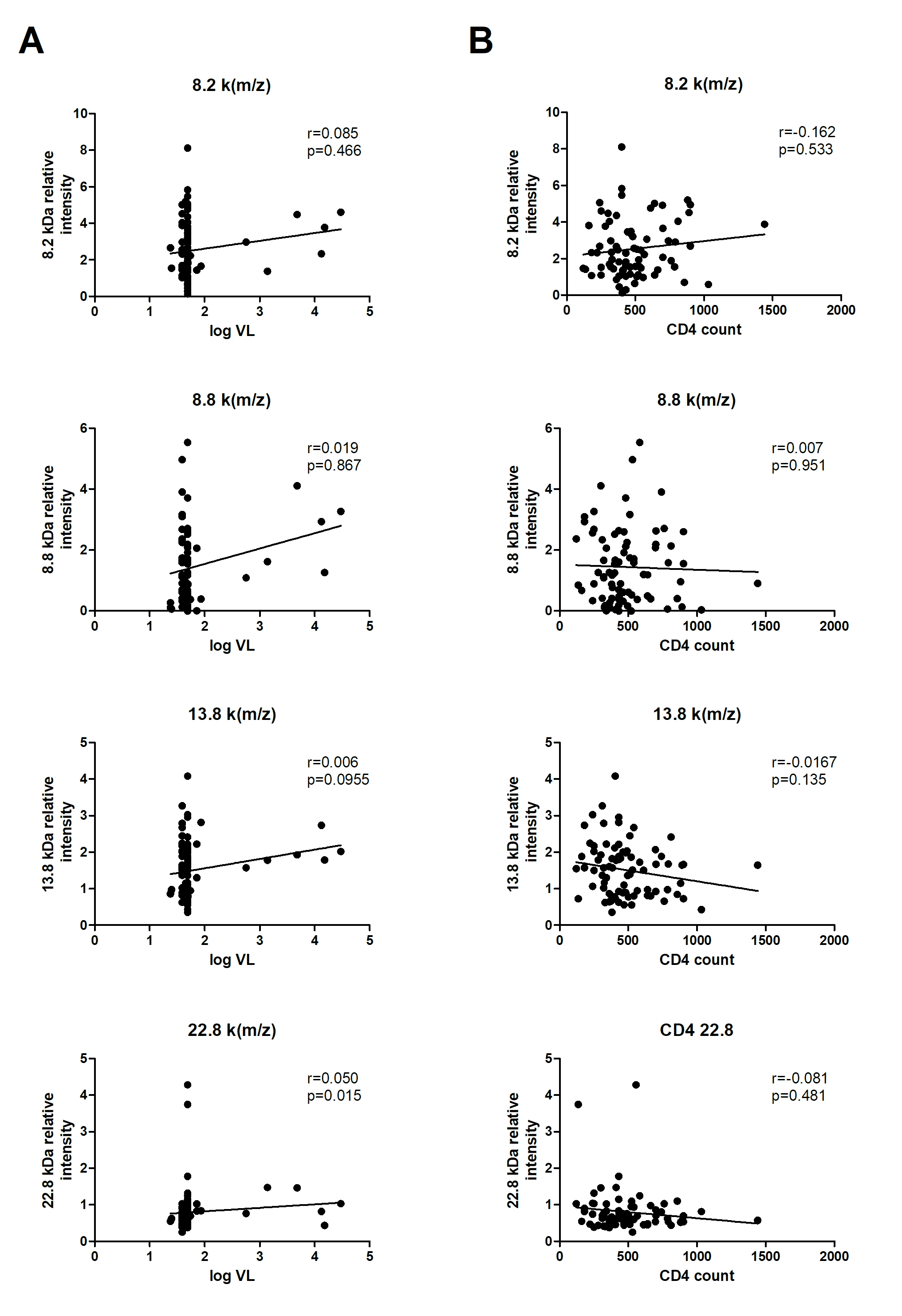
**
